# Preoperative balloon pulmonary angioplasty for chronic thromboembolic pulmonary hypertension led to successful anesthetic management for total hysterectomy under general anesthesia: a case report

**DOI:** 10.1186/s40981-020-00359-y

**Published:** 2020-07-17

**Authors:** Tomoko Ota, Kazumi Kawai, Tomoko Niihata, Hideki Fukuda

**Affiliations:** Department of Anesthesiology, Hiroshima Prefectural Hospital, 1-5-54 Ujinakanda, Minami-ku, Hiroshima-shi, Hiroshima, 734-8530 Japan

**Keywords:** Balloon pulmonary angioplasty, Chronic thromboembolic pulmonary hypertension, Pulmonary hypertension

## Abstract

**Background:**

Chronic thromboembolic pulmonary hypertension (CTEPH) is a disease of obstructive pulmonary artery remodeling as a consequence of major vessel thromboembolism. Balloon pulmonary angioplasty (BPA) is an alternative treatment for patients with inoperable CTEPH. We report a case of CTEPH which improved following preoperative BPA intervention, allowing total hysterectomy to be performed.

**Case presentation:**

A 48-year-old woman was transferred to our hospital to undergo total hysterectomy for endometrial cancer. She developed pulmonary embolism 7 months ago at another hospital, and a diagnosis of CTEPH was made based on multiple pulmonary emboli and pulmonary hypertension at our institute. Two BPA sessions for seven branches of the bilateral pulmonary arteries were conducted, resulting in a decrease of mean pulmonary artery pressure from 54 to 33 mmHg. Total hysterectomy was successfully performed under general anesthesia without any complications.

**Conclusions:**

BPA could be effective for reducing PH in patients with CTEPH undergoing noncardiac surgery.

## Background

Chronic thromboembolic pulmonary hypertension (CTEPH) is defined as the persistence of thrombi and vascular remodeling in pulmonary circulation after an embolic event associated with a mean pulmonary artery pressure (mPAP) of ≥ 25 mmHg [1, 2]. Pulmonary endarterectomy (PEA) is the only established treatment for advanced CTEPH [2]. Recent reports have suggested that balloon pulmonary angioplasty (BPA) is an alternative therapy for inoperable patients with CTEPH [3, 4]. However, there are few reports on the effectiveness of preoperative BPA for CTEPH [5].

We present a patient with CTEPH who underwent preoperative BPA to improve severe pulmonary hypertension (PH) and thereby tolerated total hysterectomy under general anesthesia.

Written informed consent was obtained from the patient’s guardian for publication of this case report and accompanying images.

## Case presentation

A 48-year-old woman (height 165 cm, weight 67 kg) had been hospitalized for schizophrenia in a psychiatric hospital. She had been prescribed sultopride hydrochloride, risperidone, and zotepine. Seven months prior to surgery, she was found to have developed dyspnea and palpitation on exertion, and lower leg edema. Computed tomography (CT) revealed thrombi in the main trunk of the left pulmonary artery as well as in the peripheries of the bilateral pulmonary arteries, confirming the diagnosis of pulmonary embolism. Transthoracic echocardiography showed a dilated right ventricle (RV) with moderate tricuspid valve regurgitation (TR) and an estimated mPAP of 43 mmHg, indicating PH (Table 1). Anticoagulant therapy was started with apixaban.

**Table 1:**
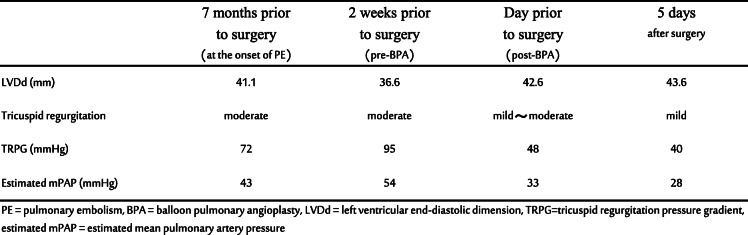
Data from transthoracic echocardiography studies

Five months prior to surgery, anticoagulant therapy was discontinued because of irregular genital bleeding, but it was resumed 2 weeks subsequently because of the reappearance of pulmonary embolism symptoms. Six weeks prior to surgery, anticoagulant therapy was again temporarily discontinued as a result of increased genital bleeding. She was diagnosed with endometrial cancer, and total hysterectomy was scheduled.

**Fig. 1:**
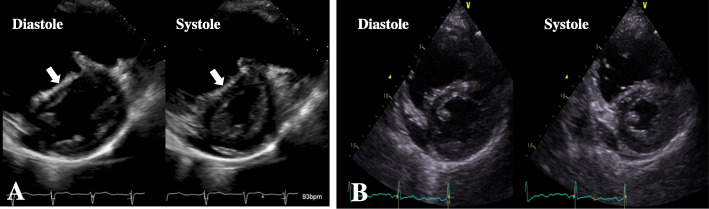
Transthoracic echocardiography before (**a**) and after (**b**) balloon pulmonary angioplasty. Flattening interventricular septum (arrow) was improved after balloon pulmonary angioplasty

**Fig. 2:**
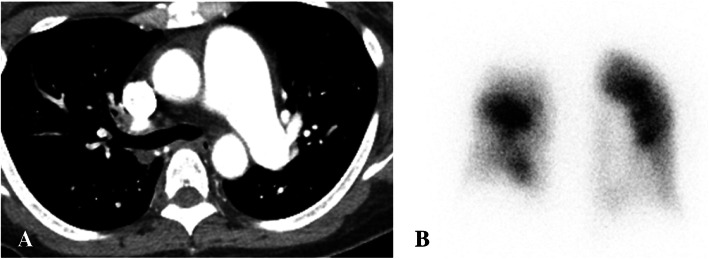
Pre-balloon pulmonary angioplasty images. **a** Contrast-enhanced computed tomography showed no emboli in the pulmonary arteries. **b** Lung perfusion scan showed multiple blood flow defects

Two weeks prior to surgery, she was transferred to our institution from the psychiatric hospital. Her vital parameters at admission were as follows: heart rate of 72 beats/min, blood pressure of 124/86 mmHg, and percutaneous oxygen saturation (SpO_2_) of 92% under oxygen administration of 2 L/min. She had severe edema of the lower extremities and jugular vein distention. Transthoracic echocardiography showed a dilated RV with flattening of the interventricular septum and with moderate TR, in addition to an estimated mPAP of 54 mmHg, suggesting severe PH (Fig. 1a). The left ventricle (LV) appeared D-shaped with preserved systolic function and exhibited an ejection fraction of 66%. Although CT showed no emboli in the pulmonary trunk (Fig. 2a), multiple blood flow defects revealed by lung perfusion scan suggested multiple emboli in the distal pulmonary artery (Fig. 2b), leading to the diagnosis of CTEPH, and promoted physicians to perform BPA in order to decrease mPAP before surgery.

Two BPA sessions were performed for bilateral pulmonary arteries by interventional cardiologists in another expert institution over a span of 6 days. The first BPA for A1, A2, A3, and A9 was performed, and the second BPA for A8b, A10a, and A10b was performed under local anesthesia. After BPA, SpO_2_ increased from 86 to 91%. Transthoracic echocardiography performed 2 days after the second BPA showed an improvement in the deformity of the LV and mild TR, and the estimated mPAP decreased to 33 mmHg (Fig. 1b). After the first BPA, warfarin was started and stopped the day before surgery, and preoperative bridging with heparin was initiated. We could not assess whether the subjective symptoms and exercise capacity of the patient improved after BPA, since she had stayed in bed all day because of depression in schizophrenia.

Total hysterectomy was performed 3 days after the second BPA. General anesthesia was selected because of the patient’s unstable mental status and perioperative anticoagulant therapy. Upon entering the operating room, her heart rate was 66 beats/min, blood pressure was 128/82 mmHg, and SpO_2_ was 92% under oxygen administration of 2 L/min. General anesthesia was induced using midazolam 3 mg, continuous infusion of remifentanil 0.1 μg/kg/min, and fentanyl 200 μg. After administration of rocuronium 40 mg, tracheal intubation was performed. Anesthesia was maintained using desflurane 3–4% in a 45% oxygen–air mixture with continuous infusion of remifentanil 0.1–0.2 μg/kg/min. A pulmonary artery catheter (PAC) was inserted in the right internal jugular vein. Transesophageal echocardiography (TEE) showed RV enlargement with mild TR and shape distortion of the interventricular septum during systole.

Introducer sheaths were placed in the right femoral artery and vein to allow for venoarterial extracorporeal membrane oxygenation in preparation for pulmonary hypertensive crisis. Hypoxia, hypercapnia, acidosis, hypothermia, and inadequate analgesia were avoided to prevent increase of pulmonary vascular resistance [6]. At 10 min after hysterectomy, the patient’s blood pressure decreased to 58/35 mmHg, with a pulmonary artery pressure (PAP) of 28/19 mmHg. TEE indicated that there was no observable change in the size of the RV and LV cavity with mild TR; therefore, we determined that the patient was not in a pulmonary hypertensive crisis. The cause of the temporary hypotension was unclear; however, it was restored rapidly after administration of a total of 12 mg ephedrine. Thereafter, values ranged 90–120/50–60 mmHg for blood pressure, 30–40 mmHg for mPAP, and 1.9–2.4 L/min/m^2^ for cardiac index.

No vasopressor other than ephedrine was required. The operation time was 1 h and 36 min and blood loss was 88 g. Because of concerns about postoperative restlessness, the TEE probe, PAC, and introducer sheaths were removed before awakening. The patient was promptly awakened and the endotracheal tube was removed. Effective postoperative analgesia was achieved with intravenous patient-controlled analgesia using fentanyl. Postoperatively, the patient was managed in the general gynecology ward. On the fifth day after surgery, anticoagulant therapy was restarted with apixaban. Transthoracic echocardiography showed mild TR, with an estimated mPAP of 28 mmHg and no worsening of PH (Table 1). The patient was transferred to the psychiatric hospital on the 19th day after surgery.

## Discussion

CTEPH is a class of PH characterized by chronic obstruction of pulmonary arteries due to the formation of thrombi. Apart from other types of PH, PEA is the only established and potentially curative treatment for CTEPH [2]. BPA is an alternative therapy for those ineligible for PEA because of comorbidities, surgically inaccessible lesions, and residual or recurrent PH after PEA [3, 4, 7, 8]. BPA is usually performed by a catheter inserted from the right jugular or femoral vein under local anesthesia. Intraprocedural complications are perforation, dissection of the pulmonary artery, and pneumothorax related to jugular vein injury. Notably, we should be careful of reperfusion pulmonary injury occurring within 48 h after BPA with an incidence of 0.3–7% [9–13], resulting from mechanical injury caused by perforation of the pulmonary artery [7]. Although BPA is effective for decreasing mPAP by approximately 20 mmHg, and if successful, to below 30 mmHg [3, 4, 7], repeated procedures, usually for 3–6 times, are required [4, 7, 8]. Therefore, BPA is not an appropriate preoperative intervention for emergency operations. Patients with contrast medium allergy or severe renal failure are not eligible. After BPA, mPAP was below 30 mmHg at follow-up of 425 days [3]. Our patient was not suitable for PEA because of the presence of lesion in the distal pulmonary arteries. After BPA, mPAP reduced from 54 to 33 mmHg and total hysterectomy was performed under general anesthesia without an increase of PAP.

BPA is an emerging alternative therapy that has been mainly developed in Japan and performed in specialized major centers; therefore, BPA is not yet widely known. Anesthesiologists should have better knowledge of BPA as a preoperative intervention for CTEPH, and they should recommend BPA to patients and surgeons as a preoperative option based on the patient’s condition and surgical procedure. The prognosis of CTEPH has been reported to be poor when mPAP is over 30 mmHg [14]. Although the degree of PH tolerable for surgery under general anesthesia is unknown, noncardiac surgery could be performed without any complications by reducing mPAP to 30 mmHg using BPA, which could in turn improve postoperative outcomes.

In conclusion, BPA has become a promising technique for the treatment of patients with CTEPH who are ineligible for PEA. BPA could be an effective preoperative option for reducing PH in patients with CTEPH undergoing noncardiac surgery.

## Data Availability

Data relevant to this case report are not available for public access because of patient privacy concerns.

## Abbreviations

BPA: Balloon pulmonary angioplasty
CT: Computed tomography
CTEPH: Chronic thromboembolic pulmonary hypertension
LV: Left ventricle
mPAP: Mean pulmonary artery pressure
PAC: Pulmonary artery catheter
PAP: Pulmonary artery pressure
PEA: Pulmonary endarterectomy
PH: Pulmonary hypertension
RV: Right ventricle
SpO_2_: Percutaneous oxygen saturation
TEE: Transesophageal echocardiography
TR: Tricuspid valve regurgitation
